# Supplementary material for: Vertically transmitted rhabdoviruses are found across three insect families and have dynamic interactions with their hosts
Source: Proc Biol Sci. 2017 Jan 25;284(1847):20162381. doi: 10.1098/rspb.2016.2381 (PMC5310039; doi:10.1098/rspb.2016.2381)
Supplement: Supplementary tables S1-6 [file rspb20162381supp1.docx]

| Primer | Sequence 5’ – 3’ |
| --- | --- |
| DImmSV_N_ F1 | CTAGCATTTGCCGGGATAAA |
| DImmSV_N_R1 | AATGCATTCCTGGTCTTTGG |
| DImmSV_L_F376 | ACAGATGCCGCCAAGATTAC |
| DImmSV_L_R1085 | AAGCTGGAGAAGATGGCAAA |
| CCapSV_L_433F | TCTGTGTCGAGAACCGAGTG |
| CCapSV_L_1183R | ACAAGCCTTGAGCTTGCATT |
| CCapSV_N_96F | TGAATTCCCCATTGATTGGT |
| CCapSV_N_809R | TCAAGTTCTTGGCCGACTCT |
| PAegRV_N_472F | ACACCTAGTCAGCCGCAGAT |
| PAegRV_N _1213R | TGCCAGAGGAGACTGGTTCT |
| PAegRV_L_F658 | ATATCCACAGCTGGCAAAGG |
| PAegRV_L_R1582 | GCCGTATGGTCCCTAACTCA |

**Table S1. PCR primers.** PCRs were carried out using a touchdown PCR cycle (95°C 15sec, 62°C (-1°C per cycle) 15sec, 72°C 1min; for 10x cycles followed by; 95°C 15sec, 52°C 15sec, 72°C 1min; for a further 25x cycles), then 72°C 4min.

| **Virus** | **Constant**  **(all pops)** | **Exponential**  **(all pops)** | **Constant**  **(one pop)** | **Exponential**  **(one pop)** |
| --- | --- | --- | --- | --- |
| CCapSV | 76 (21-140) | 51 (18-96) | 58 (19-120) | 45 (16-85) |
| PAegRV | 309 (105-588) | 218 (94-398) | 213 (69-384) | 155 (68-285) |
| DImmSV | 58 (13-107) | 16 (5-31) | 33 (6-66) | 12 (4-25) |

**Table S2. Estimates of root age under different demographic models in BEAST.** Estimates are in years, values in brackets are 95% CI’s. Models were run with viral sequences from all sampling locations or a single location (the population with the largest sample size).

| **Model** | **Log Marginal Likelihood** |
| --- | --- |
| CCapSV Morocco constant population size  CCapSV Morocco exponential growth  DImmSV Derby constant population size  DImmSV Derby exponential growth  PAegRV Hildersham constant population size  PAegRV Hildersham exponential growth | -2077.12  -2069.99  -1500.92  -1494.34  -2570.18  -2571.48 |

**Table S3. Log marginal likelihoods from path sampling analysis for different demographic models in BEAST.** Preferred models for each virus are highlighted in red.

| Location | Infected | Total tested | Proportion infected |
| --- | --- | --- | --- |
| Morocco | 13 | 64 | 0.20 (0.11-0.32) |
| Crete | 6 | 179 | 0.03 (0.01-0.07) |

**Table S4. CCapSV prevalence.** We did not have information on the sex of the medflies from Morocco, for the six infected individuals from Crete the sex ratio of infected flies was 50:50. Values in brackets are 95% confidence intervals estimated from a binomial distribution [53]

| Location | Infected | Total tested | Proportion infected |
| --- | --- | --- | --- |
| Porto, Portugal | 13 | 90 | 0.14 (0.08-0.23) |
| Chambon, France | 2 | 19 | 0.11 (0.01-0.33) |
| Falmouth, UK | 26 | 76 | 0.34 (0.24-0.46) |
| Kent, UK | 58 | 95 | 0.61 (0.51-0.71) |
| Cambridge, UK | 25 | 87 | 0.29 (0.20-0.39) |
| Coventry, UK | 23 | 69 | 0.33 (0.22-0.46) |
| Derbyshire, UK | 47 | 91 | 0.52 (0.41-0.62) |
| Edinburgh, UK | 65 | 90 | 0.72 (0.62-0.81) |

**Table S5. DImmSV prevalence.** There was no difference in prevalence between males and females (GLM, estimate=-0.104, standard error= 0.157, *z* =-0.659, *P* = 0.51). Values in brackets are 95% confidence intervals estimated from a binomial distribution [53]

| Location | Infected | Total tested | Proportion infected |
| --- | --- | --- | --- |
| Dorset, UK | 10 | 18 | 0.56 (0.31-0.78) |
| Yorkshire, UK | 19 | 24 | 0.79 (0.58-0.93) |
| North Cambridgeshire, UK | 7 | 15 | 0.47 (0.21-0.73) |
| Oxford, UK | 19 | 21 | 0.90 (0.70-0.99) |
| Somerset, UK | 16 | 21 | 0.76 (0.53-0.92) |
| South Cambridgeshire, UK | 36 | 38 | 0.95 (0.51-0.82) |

**Table S6. PAegRV prevalence.** There was no difference in prevalence between males and females (GLM, estimate=-0.226, standard error= 0.327 *z* =-0.693, *P* = 0.49). Values in brackets are 95% confidence intervals estimated from a binomial distribution [53]. We were not able to reliably estimate prevalence from Corsica and Sardinia as sequences were derived from a single offspring from each infected family.
